# Is Self-Efficacy Related to the Quality of Life in Elite Athletes after Spinal Cord Injury?

**DOI:** 10.3390/ijerph182010866

**Published:** 2021-10-15

**Authors:** Agata Goraczko, Alina Zurek, Maciej Lachowicz, Katarzyna Kujawa, Grzegorz Zurek

**Affiliations:** 1 Department of Biostructure, Wroclaw University of Health and Sport Sciences, 51-612 Wroclaw, Poland; agagoraczko@gmail.com (A.G.); maciej.lach93@gmail.com (M.L.); katarzyna.kujawa@awf.wroc.pl (K.K.); 2 Clinic of Neurorehabilitation, 54-519 Wroclaw, Poland; 3 Institute of Psychology, University of Wroclaw, 50-527 Wroclaw, Poland; alina.zurek@uwr.edu.pl

**Keywords:** quality of life, self-efficacy, spinal cord injury, elite athletes, sport

## Abstract

Background: A spinal cord injury (SCI) is a traumatic event that affects every aspect of life: physical, mental, economic, and social. The main aim of this study was to investigate self-efficacy, quality of life, and their correlations among outstanding athletes who have suffered spinal cord injuries, and to determine whether these individuals have specific psychological characteristics that contribute to a better quality of life. Methods: The study involved nine athletes with at least national-level achievements in sports prior to an SCI. Participation in the study consisted of an interview via an online communicator, followed by an online questionnaire consisting of a personal questionnaire and two scales: The World Health Organization Quality of Life Scale (WHOQoL-BREF), and the General Self-Efficacy Scale (GSES). Results: Spearman’s correlation showed a correlation between general self-efficacy, perception of quality of life, and satisfaction with own physical health, as well as psychological resources and environmental support. Conclusions: Involvement in an environment that was important to the injured person before the accident, in either a passive (in the absence of functional capacity) or active form, promotes a greater sense of self-efficacy and good QoL, regardless of the time that has passed since the accident, and despite high levels of pain or secondary health issues. To fill the gap in professional long-term healthcare services for athletes after SCIs, intervention programs should be considered that support self-efficacy, which is an important factor that can be subject to improvement.

## 1. Introduction

There is no question that spinal cord injury (SCI) is a traumatic experience causing a complete change in one’s life. In terms of physical health, the injured person not only experiences limitations in sensory and motor functions, but also a range of other disorders related to the basic functions of the urinary, digestive, respiratory, and cardiovascular systems, as well as sexual activity, sleep, spasticity, and chronic pain [1,2,3,4,5]. Work activities are often disrupted, resulting in economic decline and social isolation [6]. There have been numerous studies on the consequences of SCIs—including psychological problems—and rehabilitation programs aimed at managing the effects of SCIs [7,8,9,10,11,12]. However, the benefits of these interventions are limited due to the extent of the challenges posed by SCIs, along with the associated social disadvantages and chronic pain [13,14]. De Roon-Cassini (2009) points out that what matters most is not the physical limitation of a person after their SCI, but how the impairment is perceived by the injured person [15]. Individuals who feel less impaired may report a greater sense of life and derive greater value from daily activities [16]. Quality of life (QoL) among people with SCIs depends not so much on factors related to disability (e.g., completeness of core injury, degree of motor impairment) as on factors that are modifiable by therapy, such as self-efficacy (S-E) [13]. The QoL scales, which present the judgment of people about their health life status in different domains, and S-E, which assesses one’s belief about the ability to cope with a variety of difficult situations, are both largely subjective measures [17,18,19,20]. Hampton (2001) found that S-E was a very significant contributor to QoL when compared with disability variables and, irrespective of social support, people with higher levels of S-E appeared to be more satisfied with their lives than did people with low S-E [21,22]. Longitudinal studies suggest that S-E is a potential determinant of adjustment outcomes in the long term [23].

Ackery et al., (2007) indicate that the number of spinal cord injuries has been increasing in recent years, which may be due to the desire to perform increasingly extreme stunts and the level increase in competitive sports [24]. The study of Chan et al., (2016) identified six countries where sports account for more than 13% of SCIs (Russia, Fiji, New Zealand, Iceland, France, and Canada), as well as the highest risk sports of diving, skiing, rugby, and horseback riding [25,26]. Hockey, skiing, diving, and American football almost exclusively produce cervical SCI injuries, while more than half of the injuries in horseback riding and snowboarding are thoracic or lumbosacral injuries [25].

The main objective of this study was to investigate the correlation between S-E and QoL in a group of outstanding athletes who have experienced spinal cord injury while being active athletes, and to determine whether they have specific psychological resources that influence a better quality of life [27]. The results provide insight into the unique world of elite athletes, potentially contributing to a better understanding of whether such people with intrinsic personal attributes may show resilience related to SCIs [28].

## 2. Materials and Methods

Participation in the study consisted of an interview via an online communicator, followed by an online questionnaire consisting of a personal questionnaire and two scales: The World Health Organization Quality of Life Scale (WHOQoL-BREF), and the General Self-Efficacy Scale (GSES). Before the interview, all participants (P) read a consent form—which included the title and purpose of the study, explanation of its procedures, and confidentiality rules—and then gave informed verbal acceptance of the conditions presented (Please see the Supplementary Material). All interviews were conducted by the first author, who has years of experience both in clinical work with patients after SCIs and as their assistant. The interview was semi-structured, wherein the first part was conducted using the dialogue method, allowing the respondent to speak freely, while the second part included questions about motivation, goals in life, and social activities, among others. Each interview lasted approximately 1.5–2.5 h, was recorded and then transcribed, and its content was used to analyze the results. Consent to conduct the research project was obtained from the Senate Research Ethics Committee of the University School of Physical Education in Wroclaw, Poland (corresponding ethical approval code: 37/2018, art.27, Dz.U.1997, poz.553).

The following eligibility criteria were adopted for the study: sports achievements at the minimum national level (winning a medal at national competitions) before SCI, spinal cord injury (tetraplegia or paraplegia), and consent to participate in the study. An additional criterion was the knowledge of either the Polish or English language at a level allowing the respondent to communicate.

The personal questionnaire included questions about the participants’ demographic aspects (gender, nationality, age, marital status), injury (circumstances of injury, level of spinal cord injury, level of pain experienced daily), and sport practiced (type, best sports performance, sports activity after SCI).

Pain experienced daily was assessed using the 0–10 Numerical Rating Scale of Pain (0 = no pain, 10 = most intense pain).

Quality of Life was examined using the abbreviated version of the WHOQoL scale (WHOQoL-BREF). Adaptations of national scales were used. This scale is currently considered to be the most appropriate instrument for assessing the quality of life in people with SCIs [29,30]. Each item is described by a five-level Likert scale, where participants indicate satisfaction (5: strongly agree, 4: agree), neutrality (3: neither agree or disagree), or dissatisfaction (2: disagree, 1: strongly disagree). The two first items are examined separately and inform the researcher about one’s overall perception of one’s quality of life and health satisfaction. For the first question (Q1) of the questionnaire: “How would you rate your QoL?”, participants who answered “very poor”, “poor” or “neither poor nor good” were classified as having a negative perception of QoL, while those who answered “good” or “very good” were classed as positive. The next 24 questions describe 4 domains: physical health (D1), psychological (D2), social (D3), and material aspect (D4) [31]. For the analysis of the WHOQoL-BREF results, the raw point values obtained for the individual domain were recalculated on a scoring scale ranging from 4 to 20, in line with the World Health Organization recommendations [32]. The results are scaled in a positive direction—the higher the score, the higher the respondent’s quality of life in each domain [31].

The General Self-Efficacy Scale (GSES) is a 10-item psychometric scale that assesses optimistic self-beliefs to cope with a variety of difficult demands in life, as well as own ability. For example, item 1 is phrased: “I can always manage to solve difficult problems if I try hard enough”. The scale was created by Matthias Jerusalem and Ralf Schwarzer in 1981 and has been widely used in numerous studies worldwide [19]. Each item is rated on a four-point scale, where 1 = not at all true, and 4 = exactly true. Responses to the 10 items are summed to produce a total score, ranging from 10 to 40 points, where a higher score indicates higher self-efficacy [33]. Unlike other scales that assess optimism, this one specifically addresses personal agency.

The study conducted was qualitative. In order to deepen the analysis, calculations were performed to extract common features and correlations. This was possible due to the homogeneity of the group of participants in terms of the adopted criteria. The mean and standard deviation were calculated separately for questions Q1, Q2, and the individual domains of WHOQoL-BREF, GSES, and pain. The correlation between domains of QoL and S-E, as well as their relationship with pain and the number of years since the injury, were examined using Spearman’s rank correlation, with *p* < 0.05 indicating statistically significant results. All calculations were performed using Statistica version 13.1 in the Biostructure Research Laboratory of Wroclaw University of Health and Sport Sciences (certificate ISO 9001).

## 3. Results

### 3.1. Participants

**Figure 1:**
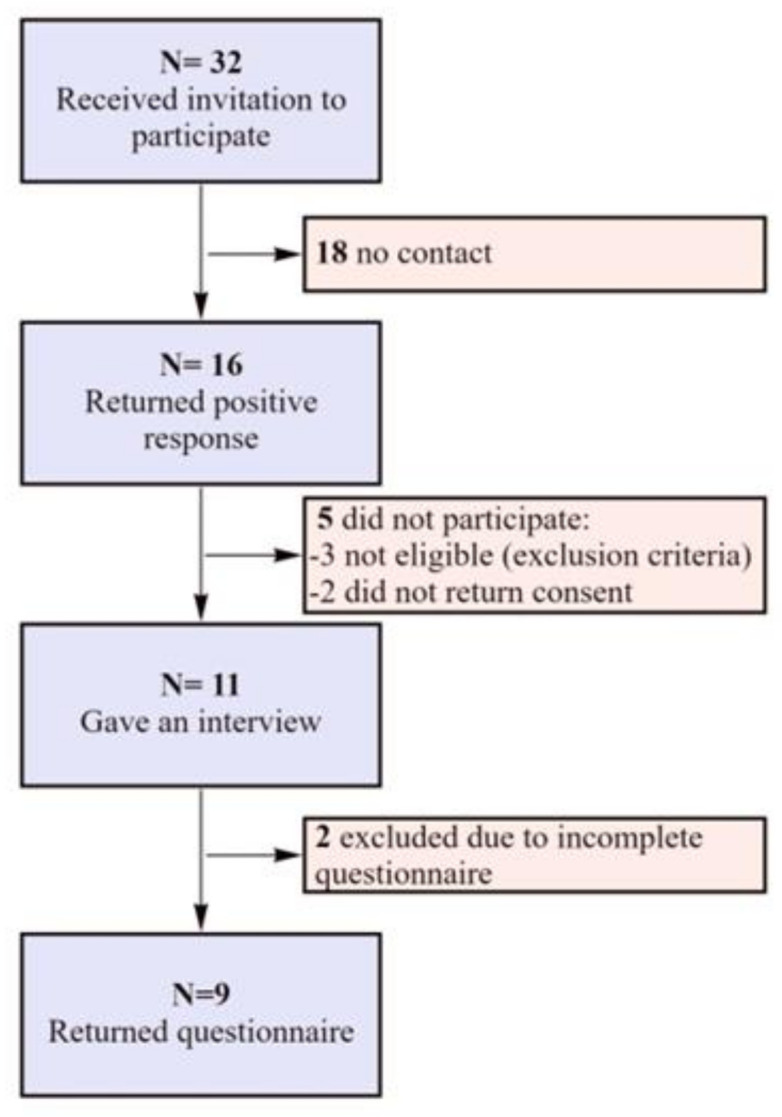
Flowchart of participant enrollment.

Figure 1 shows a flowchart of participants’ inclusion in the study. After analyzing information about spinal cord injuries among prominent athletes, and selecting individuals who met the study criteria, an invitation to participate in the project was sent by e-mail to 32 athletes from 5 continents who had suffered spinal cord injuries, from the following countries: USA, UK, Canada, Brazil, Poland, Austria, Australia, Japan, and South Africa. Ultimately, nine participants from Europe and North America participated in the study.

Table 1 lists demographic and injury-specific information on the participants. The age of the participants ranged from 24 to 55 years. Participants were selected from among both tetraplegic and diplegic patients. All participants were successful, at a minimum, at a national level before the accident, with three participants being world champions and one a European champion. Three subjects did not participate in sports after their SCI, for two of whom this was due to the amount of damage and lack of functional capacity. After their accidents, six subjects participated in sports, competing in national and international competitions, and two of them became Paralympic champions.

### 3.2. Quality of Life and Self-Efficacy

Table 2 shows the participants’ scores on each scale and its components, along with the mean and standard deviation. For the overall quality-of-life question, the mean value was Q1 = 4.11, which is a positive rating. Only two participants (P1, P4) rated their quality of life negatively; these two individuals were also not satisfied with their health status. In a comment on the WHOQoL-BREF scale, participant P1 indicated that he had been in poor health for the past 4 weeks, which is the period referred to by the scale questions. Despite the severe pain experienced by participant P1 daily, according to his response in Q5, this pain does not prevent him from doing what he needs to do. The lowest scores in all domains of quality of life as well as the GSES scale were obtained by participant P4, who indicated in his interview that he already had a depressive personality before the accident, which worsened after the SCI. It is puzzling that participant P8 had one of the lowest scores on the self-efficacy scale, despite his high sporting achievements after the accident—that is, winning a gold medal at the Paralympics and silver twice at the world championships. At the same time, participant P9, who is a three-time Paralympic gold medalist and a five-time world champion in hand-cycling, had the highest GSES score. There were no statistically significant differences in the WHOQoL-BREF and GSES scale scores among post-injury athletes (*n* = 6) and non-athletes (*n* = 3), so no such division was used in the statistical analysis.

Spearman’s correlation showed that self-efficacy was related to the general perception of quality of life (Q1) and satisfaction with one’s physical health (Q2), as well as psychological resources (D2) and environmental support (D4) (Table 3). In contrast, it is interesting that neither pain nor time since the accident was significant for the QoL or GSES measures. In the context of the specificity of the group after spinal cord injury, the lack of correlation between self-efficacy and self-assessment of physical health (D1) and social relationships (D3) is noteworthy.

## 4. Discussion

In this study, we presented the quality of life and self-efficacy of nine top athletes after SCIs, and at the same time attempted to search for the correlations between these measures. The relationship between quality of life and self-efficacy has been widely studied [13,21,22,23,27]. However, to the best of our knowledge, this study is the first to assess this aspect among elite athletes. We hypothesized that outstanding athletes, despite spinal cord injury, possess special intrinsic personal attributes that translate to good quality of life and high self-efficacy. This hypothesis is line with the findings of Kopp’s (2018) meta-analytical investigation, which showed that high emotional intelligence correlates with high athletic achievement [34,35,36].

According to previous studies, individuals with SCIs had poorer quality of life and lower self-efficacy compared to the general population [12,18,23,27]. However, in other research among athletes, higher quality of life was declared by people with spinal cord injuries participating in sport more often and at a higher level [37]. In a study by Ciampolini (2017), who evaluated the quality of life among Brazilian wheelchair tennis athletes, higher perceptions in the physical domain and total QoL were found among an elite group [38]. Despite the traumatic accident and the necessity of a complete change of lifestyle—two subjects had to give up sport completely, and six had to give up their previous sport—the participants evaluated their quality of life positively.

Undoubtedly, spinal cord injury affects physical health and, thus, influences the low scores in this domain. Participant P1 gave the lowest score in the overall assessment of his health due to the presence of decubitus ulcers and the need to remain in bed during the study period, which also influenced some of the lowest scores in the psychological domain and the deterioration of social relationships. The remaining participants (except for P3 and P4), despite para- and tetraplegia, were generally satisfied with their health. There was also no correlation found between the amount of core damage and the results of the WHOQoL-BREF and GSES scales. This confirms the findings of previous studies, where it was shown that health satisfaction is influenced more by secondary health issues than by primary accident-related damage [15,21,23,27].

According to previous studies, pain has a strong impact on quality of life [1,2,39]. However, in our study, there was no correlation between pain and QoL. Furthermore, participant P9, with one of the highest scores for pain experienced daily, also had the highest scores on the quality of life scale.

Previous studies have indicated that major problems after SCIs include social disadvantages arising from the impairment and social participation restrictions [8,13]. In Unver’s (2015) study of wrestlers at different athletic levels, the national-level wrestlers achieved the highest scores in the social domain [40]; the author explains this result by pointing to the contacts national wrestlers have with athletes from different countries through competitions. Similar findings were observed among athletes participating in the study presented here who, because of their high sporting achievements before the accident, could count on the support of the sporting community, fans, and participation in sporting life in both active (sport for the disabled) and passive (motivational speeches, role of coach) form, making them active in the social domain.

P4 had the lowest scores on the QoL scale, self-efficacy assessment with high levels of pain, and the shortest time since the accident. Depressive tendencies were also noted in the interview, as indicated by the respondent himself. In studies by Kennedy et al., (2008) and Diemen et al., (2017), higher scores for depression and anxiety were correlated with lower scores for perceived resourcefulness and self-efficacy [41,42]. According to Middleton (2007), people with negative thinking may be at higher risk of negative medical outcomes, such as pain [13]. Furthermore, patients who reported their pre-injury personality as having been depressive presented less adjustment to their SCI [23,43].

Studies by Sklett et al., (2018) and Treasure (1996) indicate that self-efficacy is associated with performance in ski jumping and among wrestlers, respectively [44,45]. Despite spinal cord injury, our subjects’ GSES scores were higher compared to previous studies involving SCI patients [33].

Prior research has produced data to suggest that S-E is associated with the perception of quality of life, general health quality, and WHOQoL domains, as also indicated by the results of our study [13,21,22]. Unlike previous studies, no correlation of S-E with the level of pain was observed, which may be related to the characteristics of the study group. The findings of previous studies suggest that sports with long durations of physically intense activity are associated with increased ability to tolerate pain [46]. The participants in this study indicated sport as having played a significant role in shaping their personalities.

The participants were characterized by a long time having passed since their accidents (>4 years). This may be the reason for the lack of correlation between the GSES and psychological or social domains, as subjects had already passed various linear stages of adjustment, leading to an optimal adjustment to SCI [47]. Only one participant (P4)—with the shortest time since the accident (4 years)—appeared to have yet to finally accept his new reality and express a desire to grow following the trauma, as is also indicated by his results. According to Catalano (2011), environmental factors are a protective element, increasing the chances of adaptive adjustment [10]. This is consistent with our results, where the correlation of the GSES scores with the environmental domain of the WHOQoL scale was shown. According to Hampton’s studies (2000, 2001), regardless of social support, individuals with higher S-E levels were found to be more satisfied with their lives than those with lower S-E levels [21,22].

Previous studies on self-efficacy show that QoL among individuals with SCIs is more dependent on the attitude a person adopts than on permanent factors related to their disability—that is, the level or completeness of their impairment [15,21,23,27]. Such findings offer hope for improving the QoL of persons with SCIs, despite the lack of impact on the disability itself.

The original intention of this study was to collect a group of subjects from all over the world so that the results would be global in scope, regardless of nationality or healthcare system. Due to the specific group of subjects and the small number of individuals meeting the inclusion criteria, we were unable to fully achieve this goal, which can be seen as a limitation. However, reaching out to nine outstanding athletes with spinal cord injuries, coming from four countries located on two continents, gives some insight into the situation under study. It also seems that increasing the number of participants would allow us to observe possible differences between athletes who participate in sports after their injury and those who do not continue a professional sports career.

## 5. Conclusions

The results of this study indicate that S-E is significantly correlated with the general perception of QoL, health, and psychological resources, as well as environmental support. Sport positively influences the wellbeing of individuals after SCIs regardless of whether it was played before and currently is not, as it encourages the development of traits that allow for better adjustment. Involvement in an environment that was important to the injured person before the accident—in either a passive (in the absence of functional capabilities) or active form—promotes greater self-efficacy and good QoL, regardless of the time elapsed since the accident, and despite high levels of pain or secondary health issues.

The results of this study offer a suggestion for clinical professionals to motivate patients to undertake active rehabilitation by showing examples of outstanding athletes who, despite the shock they experienced after their injuries, were able to adapt to their new situation, resulting in a good quality of life. We also believe that intervention programs should be considered that support S-E, which is an important factor that is subject to improvement.

## Figures and Tables

**Table 1:** Study respondents’ sociodemographic and health data.

Patient	Age	Continent	Marital Status	Years Since Injury	SCI Level	Discipline before SCI	Sport after SCI
P1	41	Europe	Divorced	14	C3/4	BMX dirt jumps	No
P2	24	Europe	Informal relationship	6	Th11/12	Karate	Wheelchair dancing
P3	29	Europe	Single	5	C6/7	Ski jumping	Rugby, skiing
P4	55	North America	Married	4	C6/7	Mountain bike racing	No
P5	31	Europe	Informal relationship	15	Th6	Motocross	Car racing
P6	37	Europe	Informal relationship	16	C4/5	Rugby	No
P7	45	North America	Married	14	Th12/L1	Mountain biking	Wheelchair basketball
P8	40	Europe	Informal relationship	17	Th11	Judo	Canoe
P9	47	Europe	Single	15	L1/2	Speedway	Hand cycling

**Table 2:** WHOQoL-BREF and GSES scores.

Scale		P1	P2	P3	P4	P5	P6	P7	P8	P9	Mean ± SD
**WHOQOL**	Q1	3 *	4	4	2 *	5	5	5	4	5	4.11 ± 1.05
	Q2	1 *	4	5	2 *	5	3	5	4	5	3.78 ± 1.48
	D1	14	18	11 *	11 *	20	15	19	16	14	15.33 ± 3.24
	D2	13 *	19	15	7 *	17	15	19	15	20	15.56 ± 3.97
	D3	13 *	20	12 *	9 *	17	17	19	16	20	15.89 ± 3.83
	D4	16	17	16	13	20	17	20	13	20	16.89 ± 2.75
GSES		31	34	33	20 *	36	33	34	28 *	39	32.22 ± 4.89
Pain		7 *	3	7 *	6 *	0	0	1	3	7 *	3.77 ± 3.03

* Lowest scale scores and highest level of pain.

**Table 3:** Spearman’s rank-order correlation.

	Q1	Q2	D1	D2	D3	D4	GSES
GSES	0.786844 *	0.736262 *	0.309322	0.781181 *	0.593220	0.870334 *	
Pain	−0.545816	−0.112136	−0.435410	0.043669	0.094842	−0.233482	−0.181061
Years since injury	−0.124132	−0.209849	−0.235302	−0.110663	−0.058826	−0.042938	−0.218495

* Correlation coefficients that are significant (*p* < 0.05).

## Data Availability

The datasets used and/or analyzed during this study are available from the corresponding author upon reasonable request.

## Supplementary Materials

The following are available online at https://www.mdpi.com/article/10.3390/ijerph182010866/s1, Consent of participation.
