# Supplementary material for: Is Self-Efficacy Related to the Quality of Life in Elite Athletes after Spinal Cord Injury?
Source: Int J Environ Res Public Health. 2021 Oct 15;18(20):10866. doi: 10.3390/ijerph182010866 (PMC8535663; doi:10.3390/ijerph182010866)
Supplement: Supplementary file 1 [file ijerph-18-10866-s001.zip › ijerph-1392777-supplementary.pdf]

Consent of participation

Project title: Cognitive functions, personal resources and the quality of life of outstanding athletes after spinal cord injury.

Investigators: Phd student Agata Gorączko, professor Grzegorz Żurek

You are being asked to participate in project conducted through Academy of Physical Education in Wroclaw in Poland. After my explanation of the project purpose and the procedures to be used, we would kindly ask you for verbal agreement to participate in this project due to bioethical commision requires. You may ask any questions you have to help you understand the project.

A. Nature and purpose of project:

The goals of the procject are following:

to measure cognitive disorders, get information about purposes in life, self-efficiency, personal competences and values, emotional control, motivation to live and social activities among world-renowned athletes after spinal cord injury, as so far there is no data containing such informations. Results of such research, history of your life and activity can be very inspirational for people with physical disabilities.

B. Explanation of Procedures:

If you agree to be in this study, you will be asked to do the following:

- Take part in interview, that will be recorded by electronic device as a video
- Complete COWAT test that measures cognitive functions
- Complete personal questionnaire and 5 other standarized questionnaires and send to following email address: agagoraczko@gmail.com

C. Confidentiality:

The answers you provide on the survey and the cognitive data we collect will be confidentiall allthough some of responses, interview information and results will be used in description of project and in potential publications. Your identity as a participant in this research study will be known and may be used in publication of the results of this study.
